# Effect of an integrated community-based package for maternal and newborn care on feeding patterns during the first 12 weeks of life: a cluster-randomized trial in a South African township

**DOI:** 10.1017/S1368980015000099

**Published:** 2015-02-09

**Authors:** Petrida Ijumba, Tanya Doherty, Debra Jackson, Mark Tomlinson, David Sanders, Sonja Swanevelder, Lars-Åke Persson

**Affiliations:** 1 Health Systems Research Unit, Medical Research Council, Tygerberg, South Africa; 2 International Maternal and Child Health (IMCH), Department of Women’s and Children’s Health, Uppsala University, Akademiska sjukhuset, SE-751 85 Uppsala, Sweden; 3 School of Public Health, University of the Western Cape, Cape Town, South Africa; 4 School of Public Health, University of the Witwatersrand, Johannesburg, South Africa; 5 Department of Psychology, Stellenbosch University, Stellenbosch, South Africa; 6 Biostatistics Unit, Medical Research Council, Tygerberg, South Africa

**Keywords:** Community health workers, Counselling, Infant feeding, HIV, Educational level, Household wealth

## Abstract

**Objective:**

To analyse the effect of community-based counselling on feeding patterns during the first 12 weeks after birth, and to study whether the effect differs by maternal HIV status, educational level or household wealth.

**Design:**

Cluster-randomized trial with fifteen clusters in each arm to evaluate an integrated package providing two pregnancy and five postnatal home visits delivered by community health workers. Infant feeding data were collected using 24 h recall of nineteen food and fluid items.

**Setting:**

A township near Durban, South Africa.

**Subjects:**

Pregnant women (1894 intervention and 2243 control) aged 17 years or more.

**Results:**

Twelve weeks after birth, 1629 (intervention) and 1865 (control) mother–infant pairs were available for analysis. Socio-economic conditions differed slightly across intervention groups, which were considered in the analyses. There was no effect on early initiation of breast-feeding. At 12 weeks of age the intervention doubled exclusive breast-feeding (OR=2·29; 95 % CI 1·80, 2·92), increased exclusive formula-feeding (OR=1·70; 95 % CI 1·28, 2·27), increased predominant breast-feeding (OR=1·71; 95 % CI 1·34, 2·19), decreased mixed formula-feeding (OR=0·68; 95 % CI 0·55, 0·83) and decreased mixed breast-feeding (OR=0·54; 95 % CI 0·44, 0·67). The effect on exclusive breast-feeding at 12 weeks was stronger among HIV-negative mothers than HIV-positive mothers (*P*=0·01), while the effect on mixed formula-feeding was significant only among HIV-positive mothers (*P*=0·03). The effect on exclusive feeding was not different by household wealth or maternal education levels.

**Conclusions:**

A perinatal intervention package delivered by community health workers was effective in increasing exclusive breast-feeding, exclusive formula-feeding and decreasing mixed feeding.

Despite ample evidence on the benefits of exclusive breast-feeding (EBF) for the first 6 months of life, its practice remains poor^(^ ^1^ ^)^. In Africa only 35 % of infants under 6 months of age are exclusively breast-fed^(^ ^2^ ^)^. The low prevalence may be attributed to factors including being HIV-positive and cultural practices^(^ ^3^ ^)^. However, EBF, as compared with mixed breast-feeding, reduces the risk of HIV infection and increases the likelihood of HIV-free survival among infants born to HIV-infected mothers^(^ ^4^ ^,^ ^5^ ^)^. In South Africa, about 28 % of child deaths have been associated with HIV and AIDS while malnutrition and diarrhoeal diseases are among the top five causes of mortality in infants^(^ ^6^ ^,^ ^7^ ^)^. Yet, South Africa has one of the lowest rates of EBF in sub-Sahara Africa of about 8 %^(^ ^8^ ^)^.

Several studies on community-based home visiting newborn care packages, conducted mainly in South Asia, have demonstrated encouraging evidence of the value of integrating maternal and newborn care in community settings delivered by community health workers (CHW)^(^ ^9^ ^–^ ^11^ ^)^. This evidence is lacking in South Africa especially among the resource-constrained peri-urban settings with high HIV prevalence.

We conducted a cluster-randomized trial to assess the impact of generalist CHW delivering a community-based intervention package on a number of key outcomes related to prevention of mother-to-child transmission of HIV (PMTCT) and HIV-free survival^(^ ^12^ ^,^ ^13^ ^)^. These CHW were employed full-time and systematically supervised. The intervention consisted of scheduled home visits to pregnant and postnatal women up to 12 weeks postnatally. The primary outcomes included HIV-free infant survival and levels of exclusive and appropriate infant feeding at 12 weeks postpartum. The present paper reports on analyses of the effect of the intervention on feeding patterns from birth to 12 weeks of age, and whether the effect of the intervention varies with the mothers’ HIV status, educational level or household asset score level.

## Methods

### Participants and setting

The design was a cluster-randomized trial. Participants were recruited from June 2008 to December 2010 and data collection was concluded in July 2011. The goal of the trial was to develop, evaluate and cost an integrated and scalable home visit package delivered by CHW, targeting pregnant and postnatal women and their newborns.

The study site was a township on the periphery of Durban municipality, KwaZulu-Natal Province, with an estimated population of one million people. Informal and formal shelters, high population density and high unemployment rates characterize the township. HIV prevalence in 2010 was estimated at 41 % among women attending antenatal public health facilities^(^ ^14^ ^)^ and the infant mortality rate was estimated at 42 per 1000 live births^(^ ^15^ ^)^.

### Recruitment of participants

Each cluster was supported by one CHW who identified all eligible pregnant women and informed them about the purpose of the study. Inclusion criteria were that women had to be 17 years or older, live in the cluster, be pregnant and intellectually capable of giving consent and willing to be visited by CHW, supervisors and data collectors. CHW provided the study team with details of participants who verbally agreed to participate in the study. Data collectors visited participants in their homes to obtain informed consent.

### Home visit intervention

Thirty CHW with at least grade-10 education level were selected from their respective clusters and trained in the intervention and control packages. The fifteen CHW from the intervention clusters were trained for 10 d on home entry, motivational interviewing techniques, breast-feeding, disclosure, antenatal care, infant feeding with emphasis on exclusive breast-feeding, breast problems and diseases, interaction with newborns, baby blues and postnatal depression, and neonatal care, including danger signs in newborns and their mothers that might warrant a referral. The training was based on a manual compiled by the principal investigators drawing on several resources, including consultation with designated training authorities in South Africa and the WHO/UNICEF Breastfeeding Counselling Course^(^ ^16^ ^)^. Training was performed through role plays, demonstrations, real-life experiences and discussions. The fifteen CHW from the control clusters were trained for 2 d on documents and information needed to access state social welfare grants. The training content was based on the Social Security Training Manual.

All CHW were trained on how to use mobile phones to capture data for each visit. In addition, CHW were paid a salary as per the South African Department of Health’s CHW remuneration package (R 3500/$US 300 per month).

The intervention was delivered by CHW living in the clusters though a structured home visiting schedule. Each visit was designated to cover specific topics related to the outcomes of the study. Visits in the intervention arm included two home visits during pregnancy, one in the first 48 h after delivery, then at 3–4 d, 10–14 d, 3–4 weeks and a final visit at 8–9 weeks. All neonates with low birth weight (≤2500 g) received two extra visits during the first week. More than 98 % of the deliveries occurred at Prince Mshiyeni Memorial Hospital. Records of all births in this hospital were checked on a daily basis and CHW supervisors contacted the CHW via a mobile phone system when an intervention group participant gave birth. Attempts were made to complete all visits regardless of how many earlier visits might have been missed. CHW received feedback on their work from the supervisors during weekly supervision meetings.

### Control cluster package

CHW living in control clusters provided essential information and support to pregnant women on how to obtain state social welfare grants. Visits in the control arm included one home visit during the antenatal period and two postnatal visits at 4–6 weeks and 10–12 weeks. Women in the intervention arm were also eligible for the state social grants and could have accessed these on their own.

### Data collection

Data collectors who previously had been involved in a similar study^(^ ^17^ ^)^ received 3 d refresher training on data collection and how to use the standard operating procedures specific for data collection and interview techniques using mobile phones.

The study questionnaires were developed and adapted from those used in a previous breast-feeding intervention trial undertaken at the same site^(^ ^17^ ^)^. HIV status of the mothers was extracted from the hospital records after delivery. Interviews were conducted by data collectors blinded to arm at Prince Mshiyeni Memorial Hospital at 12 weeks of age. Mobile phones were used to register data for the 12-week assessment of infant feeding practices as well as for the other outcomes not discussed herein^(^ ^12^ ^,^ ^13^ ^)^. Information was also collected on the mothers’ educational level and on the availability of a number of household assets, which were included in a principal component analysis to create an asset score of the household. The data collectors’ manager, data quality manager and the project manager made daily data quality checks.

### Definitions of infant feeding patterns

Breast-feeding was assessed at 12 weeks with 24 h recall of nineteen food and fluid items. Infants were grouped into six feeding patterns as summarized in Table 1^(^ ^18^ ^)^.

**Table 1:** Definitions of infant feeding patterns^(^ ^18^ ^)^

Type of infant milk/food	Definition
Breast milk	Exclusive breast-feeding: Feeding the baby with breast milk or breast milk and medicines, but the baby did not receive other food (solids or liquids)
	Predominant breast-feeding: Feeding the baby with breast milk and other fluids including water, water-based drinks, fruit juices and medicines, but the baby did not receive non-human milk or solids
Formula milk	Exclusive formula-feeding: Feeding the baby with formula milk or formula milk with medicines, but the baby did not receive other foods (solids or liquids)
	Mixed formula-feeding: Feeding the baby with formula milk and other foods (solids or liquids), but no breast milk (medicines allowed)
Breast milk and formula	Mixed breast-feeding: Feeding the baby with formula milk, breast milk and other foods (solids or liquids; medicines including traditional medicines allowed)
No breast milk or formula milk	Other feeding: Feeding the baby with solids or liquids, but no formula milk or breast milk (medicines including traditional medicines allowed)

### Sample size and cluster selection

Sample size was calculated based on increasing the HIV-free infant survival from 74 % to 84 %, with 80 % power and *α*=0·05 %. Assuming an intra-cluster correlation coefficient of 0·04 for a cluster size of fifty in a completely randomized design, it was calculated that we would need fifteen clusters and hence 750 HIV-exposed infants per arm (fifty per cluster). Loss to follow-up of approximately 20 % was added to this sample size. Based on HIV prevalence among pregnant women of 40 %, our final sample size was calculated to be 120 pregnant women per cluster.

The 2001 South African census maps of the township dividing the area into ‘sub-places’ were obtained and clusters were drawn. A baseline study was conducted in order to establish the homogeneity of clusters. The clusters in the intervention and control arms were found to be similar; therefore no stratification or matching was done. A biostatistician, who had no prior knowledge of the township, randomized clusters. Thirty clusters were assigned on a 1:1 ratio by a simple computer-generated randomization to intervention and control groups. Data collectors, unaware of the participants’ allocations, conducted informed consent and data collection.

### Statistical analysis

Intention-to-treat analysis was performed^(^ ^19^ ^)^. An asset score variable was created with principal component analysis based on sixteen binary asset items, including access to electricity, source of water; ownership of a car, refrigerator, radio, television, stove, telephone/cell phone; type of cooking fuel commonly used by the household, i.e. wood, charcoal/gas, paraffin or electricity; and the number of people living in the household.

The principal component analysis was performed in order to organize the data by reducing its dimensionality with as little loss of information as possible in the total variation these variables explain. Eigenvectors (weights) were subsequently derived from the correlation matrix. The eigenvalue (variance) for each principal component indicated the percentage of variation explained in the total data. The output from the principal component analysis was a table of factor scores/weights for each variable, which was then multiplied with each asset variable and summed to derive the asset score. This asset score was then categorized into tertiles with cut-offs at the 33·33 and 66·67 percentiles, creating an asset score with three categories.

The variables assessing feeding methods, particularly EBF, were described by HIV status, socio-economic status and level of education or years of education of the mother. Pearson’s *χ* ^2^ odds ratios and Mantel–Haenszel stratum-specific odds ratios were examined where it was hypothesized that a particular variable may affect the effect of the exposure variable of interest on the outcome.

Exposure variables, potential confounders and interaction terms that were found to be significantly associated with EBF in crude and stratified analysis were forward-fitted to a logistic regression model to calculate adjusted odds ratios. The primary outcome, infant feeding, is often associated with household environmental conditions, especially water sources and also maternal education level; therefore we adjusted for these factors in our analyses. The asset score described above was used to adjust for small differences between intervention and control groups which were noted at baseline. The likelihood ratio test was used to test the overall effect of each variable in the model and those that significantly (*P*<0·05) improved the fit of the model were retained. The Wald test was used to test the significance of the odds ratios at each level within the variable. We used a robust cluster variance approach to adjust for the cluster-randomized trial design.

The present paper focuses on secondary analysis of infant feeding (feeding patterns from birth and EBF at 12 weeks of age) and the variation across important subgroups (maternal HIV status, household asset score level and maternal educational level). For the stratified analyses interaction terms (intervention×stratifying variable) were included and a *P* value <0·10 was considered significant for the interaction. Other outcomes of the study have been published elsewhere^(^ ^12^ ^)^. The statistical software package Stata 12 was used for computation and analysis. The statistician was not blinded during the analyses. However, the statistician was independent from the study team who implemented the intervention and did not review any of the data until the completion of the trial.

### Ethical considerations

The ethics review board of the Medical Research Council (EC08-002) approved the study. In addition, a Community Advisory Board comprising community stakeholders was established and served as liaison between the township residents and research staff.

## Results

### Description of intervention and control participants and trial profile

**Fig. 1:**
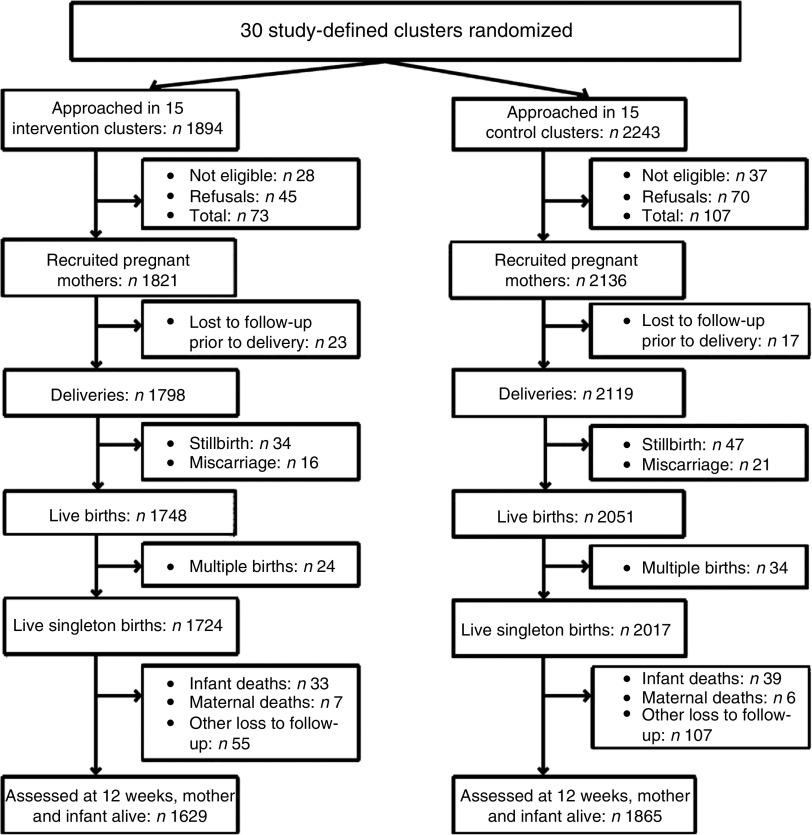
Trial profile

Between June 2008 and December 2010, a total of 1894 and 2243 pregnant women living in the intervention and control clusters, respectively, were approached in their homes for study participation by CHW. Of these, 1821 (intervention) and 2136 (control) pregnant women were deemed eligible for recruitment into the study and signed the informed consent for data collection and follow-up. At 12 weeks postpartum data were available for 1629 intervention and 1865 control live mother–infant pairs (Fig. 1).

### Baseline characteristics and effect of the intervention on infant feeding

Mothers in both arms had a median age of 23 years, the vast majority were single, and almost all had electricity in their homes and owned mobile phones. A few baseline differences were noted between the intervention and control clusters. Control clusters were marginally poorer and intervention clusters had better access to piped water in the house (Table 2). These differences were adjusted for in all analyses of feeding patterns but did not result in any change to the effect estimates.

**Table 2:** Baseline characteristics of the study participants: pregnant women aged 17 years or more from a township near Durban, South Africa, June 2008 to December 2010

	Intervention		Control	
Variable	Median or *n*/*N*	IQR or %	Median or *n*/*N*	IQR or %
Mother’s age (years)	23	20–27	23	20–27
Mother’s education level				
None	5/1629	0·3	7/1865	0·4
Primary school (1–7 years)	104/1629	6·4	129/1865	6·9
High school (8–12 years)	1418/1629	87·7	1626/1865	87·2
Tertiary (≥13 years)	102/1629	6·3	103/1865	5·5
Marital status				
Single/divorced/widowed	1429/1629	87·7	1638/1865	87·8
Married	77/1629	4·7	55/1865	3·0
Cohabiting	123/1629	7·5	172/1865	9·2
Parity				
Primipara	797/1629	48·9	869/1865	46·6
Multipara	832/1629	51·1	996/1865	53·4
Household assets				
Electricity	1152/1629	95·3	1755/1865	94·1
Refrigerator	1318/1629	80·9	1755/1865	79·8
Mobile phone	1568/1629	96·3	1787/1865	95·8
Water source				
Piped water at dwelling	996/1626	59·3	961/1865	51·5
Piped water at yard	382/1629	23·4	508/1865	27·2
Piped at public place	267/1629	16·4	361/1865	19·4
Other water source	14/1629	0·9	35/1865	1·9
Asset score				
Poorest	511/1629	32·8	634/1865	34·0
Middle	509/1629	31·3	662/1865	35·5
Wealthiest	609/1629	37·4	569/1865	30·5

IQR, interquartile range.

A total number of 1242/1629 (76 %) women in the intervention clusters and 1380/1865 (74 %) women in the control clusters introduced their babies to breast milk after birth, but only 561 (34·4 %) in the intervention clusters and 607 (32·5 %) in the control clusters initiated breast-feeding within 1 h. There was no difference in the timing of initiation of breast-feeding between study arms (Table 3).

**Table 3:** Feeding patterns in the intervention and control arms of the cluster-randomized trial evaluating the effects of community-based counselling on feeding patterns during the first 12 weeks after birth, Durban, South Africa, 2011

	Intervention		Control		Crude*		Adjusted†	
Feeding type	*n/N*	%	*n/N*	%	OR	95 % CI	OR_adj_	95 % CI
Initiation of breast-feeding within 1 h after birth	561/1629	34·4	607/1865	32·5	1·09	0·93, 1·27	1·08	0·93, 1·26
Exclusive breast-feeding, 24 h recall at 12 weeks after birth	441/1629	27·1	260/1865	13·9	2·29	1·80, 2·92	2·31	1·82, 2·93
Exclusive formula-feeding, 24 h recall at 12 weeks after birth	240/1629	14·7	172/1865	9·2	1·70	1·28, 2·27	1·70	1·27, 2·26
Predominant breast-feeding, 24 h recall at 12 weeks after birth	112/1629	6·9	77/1865	4·1	1·71	1·34, 2·19	1·70	1·33, 2·17
Mixed formula-feeding, 24 h recall at 12 weeks after birth	434/1629	26·6	652/1865	35·0	0·68	0·55, 0·83	0·67	0·54, 0·82
Mixed breast-feeding, 24h recall at 12 weeks after birth	395/1629	24·3	694/1865	37·2	0·54	0·44, 0·67	0·54	0·44, 0·67
Other feeding, 24h recall at 12 weeks after birth‡	7/1629	0·4	10/1865	0·5	0·80	0·27, 2·36	0·75	0·25, 2·27

* Adjusted for cluster.

† Adjusted for cluster, household asset score level and maternal education level.

‡ Infants who received neither breast milk nor formula milk, but most commonly cereals, porridge and bread.

The intervention resulted in significant effects on infant feeding patterns at 12 weeks postpartum: EBF was doubled, exclusive formula-feeding increased, predominant breast-feeding was also increased, and mixed formula-feeding as well as mixed breast-feeding were reduced (Table 3).

### Effect of the intervention in groups defined by HIV status or social characteristics

The effect of the intervention on EBF at 12 weeks of age was stronger in the HIV-negative group of mothers than in the HIV-positive mothers (Table 4). The reduced risk of mixed formula-feeding in the intervention arm was statistically significant only in the HIV-positive stratum.

**Table 4:** Effects of the community-based counselling intervention on infant feeding at 12 weeks of age in HIV-positive and HIV-negative groups of mothers, Durban South Africa, 2011

	HIV-negative				HIV-positive				
	Crude		Adjusted*		Crude		Adjusted*		*P* value for interaction
Feeding outcome	OR	95 % CI	OR_adj_	95 % CI	OR	95 % CI	OR_adj_	95 % CI	intervention×HIV status
Exclusive breast-feeding	2·70	1·98, 3·69	2·70	2·01, 3·70	1·70	1·30, 2·21	1·70	1·32, 2·20	0·01
Exclusive formula-feeding	1·70	1·06, 2·74	1·70	1·06, 2·74	1·93	1·32, 2·83	1·93	1·31, 2·83	0·69
Predominant breast-feeding	1·82	1·38, 2·40	1·82	1·38, 2·40	1·37	0·70, 2·70	1·36	0·69, 2·65	0·48
Mixed formula-feeding	0·83	0·65, 1·05	0·83	0·65, 1·05	0·53	0·37, 0·75	0·53	0·37, 0·74	0·03
Mixed breast-feeding	0·46	0·36, 0·58	0·47	0·37, 0·60	0·49	0·33, 0·75	0·49	0·33, 0·73	0·82

* Adjusted for cluster, household asset score level and maternal education level.

The intervention did not have any differential effects on infant feeding outcomes at 12 weeks of age in subgroups defined by household asset score levels or maternal education levels (test for interactions, all *P*>0·10; see online supplementary material, Supplemental Tables 1 and 2).

## Discussion

The present study demonstrates that an integrated package delivered by systematically supervised, remunerated, full-time CHW resulted in significant and favourable effects on infant feeding patterns at 12 weeks of age in a poor semi-urban South African area, with high HIV prevalence among mothers. The size of the association was relatively modest, but larger than in a previous comparable trial^(^ ^17^ ^)^. The positive effect on EBF was more pronounced among HIV-negative mothers, which may reflect the difficulties of current evidence-based feeding recommendations in a setting of high HIV prevalence and recent drastic changes in infant feeding policy^(^ ^20^ ^)^.

Still, more than 70 % of the mothers practised infant feeding patterns other than EBF, such as exclusive formula-feeding, predominant breast-feeding, mixed formula-feeding or mixed breast-feeding. This may indicate that individual motivational interviewing^(^ ^21^ ^)^ alone may not be sufficient to change what has become a predominantly formula-feeding culture in South Africa.

A recent systematic review indicated that studies combining individual (one-on-one counselling and support) and group counselling (counselling in groups of mothers or with other members of their families) were superior in increasing EBF and breast-feeding rates at birth, during the first month and between 1 and 5 months in low- and middle-income countries^(^ ^22^ ^)^. In our trial, we included in the motivational interviewing sessions other household members related to the mother who were willing to take part in the dialogue, including participants’ mothers, grandmothers, sisters, husbands or boyfriends. However, the trial was not designed to systematically measure the extent to which other members of the family participated in these sessions or what their contribution was^(^ ^23^ ^)^. Qualitative sub-studies in our trial showed that study participants’ mothers, friends and boyfriends were significant people who need to be included in the infant feeding decision making^(^ ^24^ ^)^. Studies in other African countries such as Ethiopia and Malawi have documented the importance of participants’ mothers and mothers-in-law in infant feeding decision making^(^ ^25^ ^,^ ^26^ ^)^.

Breast-feeding initiation rates of 76 % (intervention) *v*. 74 % (control) were similar to those reported elsewhere in South Africa^(^ ^27^ ^)^ but below the WHO target of 90 %. Surprisingly, less than 35 % of the mothers in either intervention or control clusters experienced timely initiation of breast-feeding, i.e. within 1 h after delivery, despite 98 % of the deliveries taking place in a hospital with Baby Friendly Hospital Initiative credentials. Timely initiation of breast-feeding is crucial^(^ ^28^ ^)^. A study conducted in rural Ghana in 2006 showed that initiation of breast-feeding within the first hour after birth was associated with prevention of 22 % of the neonatal deaths^(^ ^28^ ^)^. Infants initiated to breast-feeding and who exclusively breast-fed within 1 h after birth were nine times less likely to die than those who were initiated on infant formula and breast milk only within 72 h of birth^(^ ^28^ ^)^. Similarly, in Nepal 19 % of neonatal deaths were prevented with universal initiation of breast-feeding within the first hour^(^ ^29^ ^)^. Fear of HIV transmission through breast milk as well as poor or lack of support from the hospital nurses may have contributed to poor breast-feeding initiation^(^ ^30^ ^)^.

Our findings suggest that the community-based package of home visits for mothers and newborns had a significant effect on improving EBF, particularly in HIV-negative mothers. The smaller effect in HIV-positive mothers may be associated with the free provision of formula to HIV-positive mothers. In 2002 formula milk was introduced in the PMTCT programme and was distributed by health-care facilities throughout the country in order to offer HIV-positive mothers an alternative to breast-feeding to avoid postnatal HIV transmission^(^ ^31^ ^)^. As a result of this policy, many mothers may view formula milk as a better form of infant food^(^ ^24^ ^)^.

In 2011 the national Department of Health revised the infant and young child feeding guidelines to support exclusive breast-feeding irrespective of HIV status^(^ ^32^ ^)^. An exit interview with CHW who carried out the intervention revealed that none of them had exclusively breast-fed their babies although they were given the responsibility to influence behavioural change. Studies conducted in Southern Africa discuss challenges of counselling that go beyond training^(^ ^30^ ^)^. It is most likely that counsellors themselves, in this case the CHW, are not convinced that breast-feeding should be practised by HIV-positive mothers or mothers with unknown HIV status. Despite the drawbacks discussed above, the increase in EBF prevalence is substantive when compared with the rates reported in the last two South African Demographic and Health Surveys of 7 % in 1998 and 8 % in 2003^(^ ^8^ ^,^ ^33^ ^)^.

Education level and socio-economic status of the mother have been found to be associated with the prevalence of EBF, particularly in high- and middle-income country settings such as the UK and Sweden^(^ ^34^ ^,^ ^35^ ^)^. The experience in other African countries is mixed. In two studies (Nigeria and Ghana) neither maternal age nor education level was associated with practising EBF^(^ ^36^ ^,^ ^37^ ^)^, while a study in Uganda showed a positive association between EBF and maternal level of education^(^ ^38^ ^)^. However, in our study the intervention had no differential effect in groups of women defined by educational level or household asset score level.

Studies conducted in sub-Sahara Africa show that in addition to fear of HIV transmission, social factors such as community norms, the role of the significant others in breast-feeding decision making and structural factors such as single motherhood and teenage pregnancy are key factors associated with choices related to infant feeding^(^ ^39^ ^)^. Participants in our study were relatively young mothers (average age of 23 years), single or divorced (87 %) and 23 % were teenage mothers attending school. The demographic characteristics in our study population may be different from those observed in high- and middle-income countries such as the UK and Sweden^(^ ^34^ ^,^ ^35^ ^)^.

### Strengths of the study

We used a randomized design, a gold standard for testing the effectiveness of interventions in real-world settings. We recruited an adequate sample size and measured independent and similar outcomes in intervention and control areas. Data collectors performed the interviews in IsiZulu and were conversant not only with the language, but also with the norms required to ask the questions appropriately.

### Limitations of the study

Data on infant feeding behaviour are usually collected through self-reporting. Therefore there is always a possibility that participants may report what the researcher wants to hear. However, the large differences in EBF make it unlikely that the result could be attributed solely to reporting bias.

In order to limit loss to follow-up and high costs of running the study, infants were followed-up for 3 months and data were collected at a single point. Therefore, the EBF data are not suitable for analysing trends or reflecting on EBF at 6 months (the WHO recommended length of EBF). In addition, we did not include questions to indicate the ages of the infants when they were introduced to solids and other liquids, although this knowledge is crucial in developing strategies to support mothers to breast-feed. The study was conducted in a peri-urban community and therefore the results cannot be generalized to rural settings.

### Implications

EBF for the first 6 months of life is a crucial intervention for child survival. Counselling has been shown to be largely ineffective in the South African setting in improving infant feeding, especially in HIV-positive mothers^(^ ^30^ ^,^ ^40^ ^)^.

A previous cluster-randomized trial in three sites in South Africa, including the township included in the present study, demonstrated a small but significant increase in EBF from 6 to 12 % using single-purpose CHW^(^ ^16^ ^)^. To our knowledge, our study is the first cluster-randomized trial in a high HIV prevalence township in South Africa to document the impact of motivational interviewing techniques used by generalist, remunerated and systematically supervised CHW to promote EBF and appropriate infant feeding. The long history of formula milk provision through the health system coupled with cultural feeding practices makes the effective communication of strategies to improve EBF complex^(^ ^31^ ^)^. Currently South Africa is in the process of training CHW to implement community-based health-care services as part of the new re-engineered primary health-care package and our findings provide important lessons about the design of the maternal newborn care component^(^ ^41^ ^)^.

## Conclusion

Generalist, remunerated and systematically supervised CHW, who used motivational interview techniques, were effective in improving EBF, especially among HIV-negative mothers. CHW have the potential to improve EBF in similar settings in Southern Africa. Rigorous strategies are needed to communicate the policy change regarding infant feeding and HIV.

## Supplementary material

For supplementary material accompanying this paper visit https://doi.org/10.1017/S1368980015000099. click here to view supplementary material
