# Supplementary material for: Effect of an integrated community-based package for maternal and newborn care on feeding patterns during the first 12 weeks of life: a cluster-randomized trial in a South African township
Source: Public Health Nutr. 2015 Feb 9;18(14):2660–8. doi: 10.1017/S1368980015000099 (PMC4564360; doi:10.1017/S1368980015000099)
Supplement: Supplementary file 1 [file S1368980015000099sup001.doc]

**Supplemental Table 1.** Effect of the intervention on infant feeding at 12 weeks of age in mothers with less than 12 years of education and those with more than 12 years of education

|  |  | Years of education | | |  |
| --- | --- | --- | --- | --- | --- |
| Feeding outcome | Less than 12 Crude OR (95% CI) | Less than 12 OR_adj*_ (95% CI) | 12 and above Crude OR | 12 and above OR_adj*_ (95% CI) | *P*-value for interaction intervention*Years of education |
| Exclusive breast-feeding | 2.4(1.9-3.1) | 2.4(1.9-3.2) | 2.1(1.7-2.8) | 2.1(1.5-3.0) | 0.54 |
| Exclusive formula feeding | 1.8(1.4-2.7) | 1.8(1.3-2.6) | 1.6(1.2-2.2) | 1.9(1.3-2.7) | 0.60 |
| Predominant breast-feeding | 1.7(1.1-2.6) | 1.8(1.3-2.6) | 1.7(1.1-2.7) | 1.6(1.1-2.3) | 0.98 |
| Mixed formula feeding | 0.7(0.5-0.8) | 0.6(0.5-0.8) | 0.7(0.6-0.9) | 0.7(0.6-0.9) | 0.66 |
| Mixed breast-feeding | 0.5(0.4-0.6) | 0.5(0.3-0.6) | 0.6(0.5-0.7) | 0.5(0.4-0.6) | 0.30 |

*Adjusted for cluster, asset score and mother’s HIV status.

**Supplemental Table 2.** Effect of the intervention on infant feeding at 12 weeks of age in mothers in households with different levels of wealth (asset score)

|  | SES | | | | | |  |
| --- | --- | --- | --- | --- | --- | --- | --- |
| Feeding outcome | Poorest Crude OR (95% CI) | Poorest OR_adj*_ (95% CI) | Middle Crude OR (95% CI) | Middle OR_adj*_ (95% CI) | Wealthiest Crude OR (95% CI) | Wealthiest OR_adj*_ (95% CI) | *P*-value for interaction intervention*SES tertile |
| Exclusive breast-feeding | 2.7(1.9-4.0) | 2.70(1.8-4.0) | 2.4(1.8-3.2) | 2.4(1.8-3.2) | 1.8(1.3-2.7) | 1.9(1.3-2.7)| | 0.18 |
| Exclusive formula feeding | 1.4(0.9-2.2) | 1.4(0.9-2.2) | 1.9(1.3-2.9) | 2.2(1.4-3.5) | 1.8(1.2-2.7) | 1.8(1.2-2.8) | 0.43 |
| Predominant breast-feeding | 1.4(1.0-2.1) | 1.4(1.0-2.1) | 1.7(1.1-2.7) | 1.8(1.1-2.8) | 2.0(1.4-2.9) | 2.0(1.4-2.9) | 0.49 |
| Mixed formula feeding | 0.6(0.4-0.8) | 0.6(0.4-0.8) | 0.6(0.5-0.8) | 0.6(0.5-0.9) | 0.8(0.6-1.0) | 0.8(0.6-1.0) | 0.30 |
| Mixed breast-feeding | 0.6(0.4-0.8) | 0.6(0.4-0.8) | 0.5(0.4-0.7) | 0.5(0.3-0.6) | 0.5(0.4-0.7) | 0.5(0.4-0.6) | 0.82 |

*Adjusted for cluster, years of education and mother’s HIV status.
